# Aptamer-Functionalized Platform for Selective Bacterial Isolation and Rapid RNA Purification Using Capture Pins

**DOI:** 10.3390/s25061774

**Published:** 2025-03-13

**Authors:** Md Aminul Islam, Rebecca Giorno, Gergana G. Nestorova

**Affiliations:** 1 Molecular Sciences and Nanotechnology, Louisiana Tech University, Ruston, LA 71272, USA; mai012@latech.edu; 2 School of Biological Sciences, Louisiana Tech University, Ruston, LA 71272, USA; rgiorno@latech.edu

**Keywords:** RNA purification, aptamer-based biosensing, piezoelectric bacterial lysis

## Abstract

Efficient bacterial lysis and RNA purification are essential for molecular diagnostics and biosensing applications. This study presents a piezoelectric platform integrated with gold-plated RNA capture pins (RCPs) functionalized with synthetic oligonucleotides to extract and enrich *E. coli* 16S ribosomal RNA (rRNA). The 3D-printed device enables selective bacterial capture using *E. coli*-specific aptamers and incorporates a piezoelectric transducer operating at 60 kHz to facilitate bacterial cell wall disruption. The platform demonstrated high specificity for *E. coli* over *B. cereus*, confirming aptamer selectivity. *E. coli* viability assessment demonstrated that positioning the piezoelectric plate in contact with the bacterial suspension significantly improved the bacterial lysis, reducing viability to 33.68% after 15 min. RNA quantification confirmed an increase in total RNA released by lysed *E. coli*, resulting in 10,913 ng after 15 min, compared to 4310 ng obtained via conventional sonication. RCP-extracted RNA has a threefold enrichment of 16S rRNA relative to 23S rRNA. RT-qPCR analysis indicated that the RCPs recovered, on average, 2.3 ng of 16S RNA per RCP from bacterial suspensions and 0.1 ng from aptamer-functionalized surfaces. This integrated system offers a rapid, selective, and label-free approach for bacterial lysis, RNA extraction, and enrichment for specific types of RNA with potential applications in clinical diagnostics and microbial biosensing.

## 1. Introduction

The rapid genotyping of bacteria is important in medical research, food and water safety monitoring, and space exploration. In clinical settings, accurate and fast detection of bacterial pathogens is crucial for diagnosing infections, selecting effective treatments, and reducing the spread of infectious diseases [1]. In biomedical research, rapid genotyping enables the study of bacterial genetics and interactions, advancing the fields of biotechnology, drug development, and microbiology [2]. In industrial and environmental microbiology, fast purification and genetic analysis allow for the timely engineering of bacteria for applications such as bioremediation, agriculture, and the production of pharmaceuticals or biofuels [3]. Fast methods enable the analysis of large numbers of bacterial strains, which is important in drug discovery, where many strains need to be screened for specific characteristics or drug resistance [4]. In space exploration, missions to the Moon and Mars require fast, sensitive, lightweight technologies for genetic analysis with minimal liquid handling. Lab-on-a-chip technology provides better precision, compact size, and reduced payloads, making it ideal for genetic analysis in microgravity conditions [5,6].

The initial step in bacterial RNA purification involves the lysis of the bacterial cell wall, which can be performed using mechanical, thermal, chemical, and enzymatic techniques. Thermal and SDS-based methods, though effective, have several disadvantages. Thermal lysis requires high temperatures, which can degrade nucleic acids and be less efficient in the lysis of bacteria with thick cell walls [7]. The detergents used in SDS-based lysis can interfere with downstream applications, such as PCR or sequencing [8]. Mechanical lysis includes bead beating, where cells are vortexed with small beads, resulting in physical lysis through collisions. This approach was successfully integrated into a low-power device for rapid lysis [9]. Sonication uses sound waves to lyse cells through cavitation to disrupt cell structures however, it can fragment nucleic acids [10]. When an electric field is applied to piezoelectric materials, they produce mechanical stresses or vibrations that can disrupt bacterial cell walls, releasing their intracellular components [11]. The piezoelectric technique is particularly efficient for lysing bacterial species with thick cell walls. The advantages of the piezoelectric approach include detergent-free, efficient, reduced thermal degradation of nucleic acids, and easy incorporation into a microfluidic device [12].

Despite advancements in bacterial RNA purification, existing methods are often time-consuming, require specialized equipment, and are unsuitable for resource-limited or microgravity environments. There is a need for a rapid, efficient, and portable method for RNA purification that enables genetic analysis in diverse applications. The most common approaches for RNA purification from bacteria include silica-column-based and magnetic bead-based purification. Both methods offer simple and efficient bacterial nucleic acid purification but are associated with high consumables costs and the need for specialized equipment, such as magnetic racks and centrifuges [13,14]. We have developed a rapid RNA purification method for plants [15] and 3D spheroids [16] that was successfully validated for plant genetic analysis on the International Space Station [17]. At the core of the technology is a microscopic pin functionalized with synthetic oligonucleotides for the selective capture and enrichment of genetic material for downstream genetic analysis. RNA sequencing analysis of the captured RNA indicated that the purified nucleic acid was enriched for the selected target RNA type [18].

**Figure 1:**
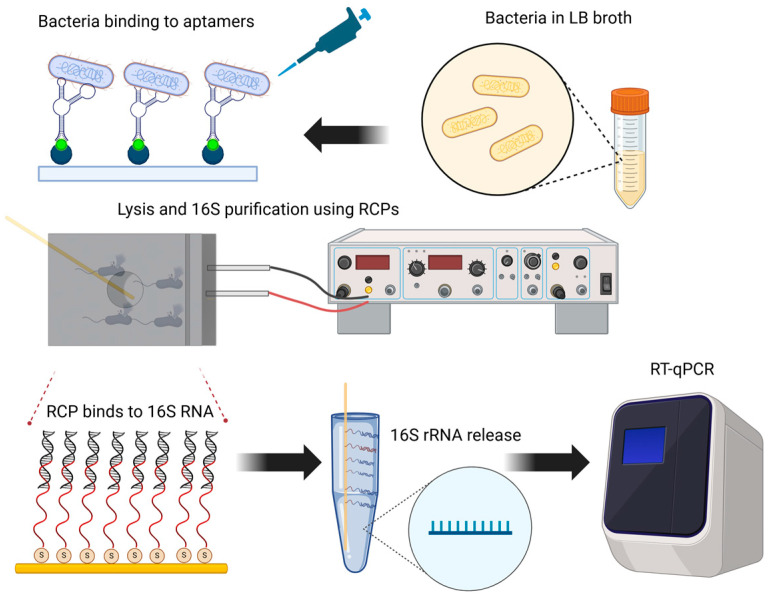
Schematic representation of the experimental workflow for the selective capture of bacteria, piezoelectric lysis, and direct purification of 16S rRNA, followed by PCR amplification and genetic analysis.

In this study, we present an RNA capture pin (RCP) technology that integrates aptamer-based bacterial capture, piezoelectric lysis, and rapid, liquidless RNA purification for downstream genetic analysis. The gold-plated microscopic pin is functionalized with synthetic oligonucleotides designed to hybridize to the 16S ribosomal RNA (rRNA) of *Escherichia coli* (*E. coli*). The platform enables selective bacterial capture using aptamers, followed by piezoelectric wave-induced cell wall disruption, eliminating the need for detergents or high temperatures. After lysis, the RCP is inserted into the bacterial suspension for two minutes to capture RNA, which is then released in RNase-free water for subsequent analysis via reverse transcription quantitative PCR (RT-qPCR) (Figure 1). Unlike conventional methods, such as magnetic bead- or column-based RNA extraction, which require multiple reagents and processing steps, our platform offers a streamlined, reagent-minimal approach with enhanced portability. This is particularly advantageous for point-of-care diagnostics and spaceflight applications, where rapid and efficient nucleic acid purification is critical. By combining highly selective aptamer binding, energy-efficient lysis, and amplification within a single platform, this technology provides a robust alternative to existing extraction techniques, facilitating on-site RNA analysis with minimal requirements for lab equipment. This nucleic acid capture method provides high RNA recovery rates and specificity for downstream genetic applications. We purified and amplified 16S rRNA from a single *E. coli* colony grown overnight in LB broth and lysed using the piezoelectric platform. The combination of piezoelectric lysis and RCP technology simplifies the overall process of bacterial genotyping, improves the speed of nucleic acid purification, and provides enrichment for specific types of RNA. This method can be applied in research and clinical settings for rapid and efficient detection and analysis of bacterial RNA.

## 2. Materials and Methods

### 2.1. Thiol-Conjugated 16S-Specific Oligos Immobilization to Gold-Plated RNA Capture Pins

Thiol-conjugated bacterial 16S-specific oligonucleotides were immobilized on Tai Chi^®^ gold-plated pins (0.20 mm × 13 mm, Lhasa OMS, Weymouth, MA, USA). Before functionalization, the gold surface was cleaned using a solution of 30% hydrogen peroxide, 25% ammonia, and Milli-Q water (1:1:5) at 80 °C for 10 min to remove organic contaminants, followed by washing with RNAse-free water [19]. The thiol-modified oligonucleotides were shipped in their oxidized form (protected by S–S bonds). Before coupling to gold, the oligos were reduced with 100× excess Tris[carboxyethyl] phosphine (TCEP 10 mM, Sigma-Aldrich, St. Louis, MO, USA, cat. #C4706) for 2 h at room temperature, according to the vendor guidelines. Thiol-conjugated synthetic oligonucleotides complementary to the *E. coli* 16S rRNA region (1541 base pairs; GenBank: J01859.1) were designed using the NEB Next Custom RNA Depletion Tool and synthesized by Integrated DNA Technologies (cat. #30712121). The RNA capture pins were incubated for 24 h at 4 °C in 200 μL of 10 μM thiol-conjugated oligonucleotide mixture. To minimize nonspecific binding, probes were incubated in 300 μM (11-mercaptoundecyl) hexa(ethylene glycol) (TOEG6) (Sigma-Aldrich, cat. #675105) for 30 min [20].

### 2.2. Scanning Electron Microscopy of RNA Capture Pin Functionalization

The stainless-steel gold-plated pins were characterized using scanning electron microscopy (SEM) to examine their surface morphology. Elemental composition analysis was performed using energy-dispersive X-ray spectroscopy (EDS). The RNA capture pins were characterized using an electron microscope (Hitachi S4800 FE-SEM, Hitachi High-Technologies, Schaumburg, IL, USA) equipped with EDS. The primary objectives were to assess the functionalization efficiency and determine the surface topology and elemental composition of the RCPs. Each group consisted of five probes, including cleaned plain pins and pins functionalized with thiol-conjugated 16S-specific oligonucleotides passivated with 300 μM TOEG6. The RCPs in each group were imaged using 3 kV voltage, 15 mm working distance, and 200× and 400× magnifications.

### 2.3. Fabrication of the Bacterial Lysis Platform with Integrated Piezoelectric Plates

The device was designed using SolidWorks (Version 2023 SP2.1) with dimensions of 35 mm × 28 mm × 10 mm and a wall thickness of 0.1 mm. The design was exported in STL format and fabricated using a Formlabs Form 4 3D printer. A transparent, biocompatible poly (methyl methacrylate) photopolymer resin (Flexible 80A, Formlabs, Somerville, MA, USA) was selected because it contained surface hydroxyl groups that enable functionalization of the inner surface of the device. 3D printing was performed at a set layer thickness of 0.1 mm. After printing, the device was rinsed in isopropyl alcohol to remove uncured resin and UV-cured at 60 °C for 15 min to ensure structural integrity. Support structures were carefully removed to preserve the thin walls. A piezoelectric plate made of PZT-5H (Steminc, Davenport, FL, USA, cat. #SMPL25W5T30311) with dimensions 25 × 5 × 0.30 mm was used as an ultrasonic transducer to generate high-frequency vibrations. The optimal placement of the plate for enhanced lysis efficiency was investigated by testing two configurations: attachment to the outer surface of the device using adhesive tape and submersion in the bacterial solution. Two wires were soldered to the plate to enable transducer functionality. An Agilent 33220A 20 MHz function generator supplied a sinusoidal signal at the plate’s resonance frequency (60 kHz), producing ultrasonic waves that induced mechanical stress in the bacterial solution. A handheld sonicator (ATO, Diamond Bar, CA, USA, cat. #ATO-HUH-5) operating at 30 kHz served as a control for lysis performance comparison.

### 2.4. Aptamers Functionalization of the Platform and Assessment of Bacterial Binding

The lower channel wall of the device was functionalized with streptavidin for covalent attachment of biotin-conjugated aptamers for selective capture of *E. coli*. The resin was treated with 10% 3-aminopropyltriethoxysilane (APTES) (Thermo Fisher Scientific, Waltham, MA, USA, #80370) in acetone for 10 min to introduce amine groups [21]. 20 mM EZ-Link™ NHS-Biotin (Thermo Fisher Scientific, cat. #20217) was added to the APTES-coated surface and incubated for 30 min. Dynabeads™ M-280 (Thermo Fisher Scientific, cat. # 11205D) confirmed successful surface modification with biotin. Streptavidin (Thermo Fisher Scientific, cat. #21121) at a concentration of 1 µg µL-1 was applied to the biotinylated surface and incubated for 25 min. The changes in surface roughness after APTES, biotin, and streptavidin immobilization were characterized using a 3D Laser Scanning Confocal Microscope (Keyence Corp., Osaka, Japan). Biotin-conjugated aptamers (100 µM) were prepared in Tris buffer (Teknova, Hollister, CA, USA, #T2075) and incubated with the streptavidin-coated surface for 35 min. The aptamer sequence was previously validated to bind to proteins on the outer cell wall of *E. coli* and has the following sequence 5′-ATCCGTCACACCTGCTCTACGGCGCTCCCAACAGGCCTCTCCTTACGGCATATTATGGTGTTGGCTCCCGTAT-3′ [22]. The bacteria were cultured in LB broth (Thermo Fisher Scientific, cat. #10855001) at 37 °C for 18 h. The cultured bacteria were introduced to the lower channel wall and incubated for 3 h to allow binding. FITC-conjugated aptamers (100 µM) were then applied to the bound bacteria for fluorescence-based visualization. *Bacillus cereus* was used as a negative control to assess non-specific binding. Fluorescence microscopy was employed to confirm and visualize the selective binding of *E. coli* to the functionalized surface.

### 2.5. Assessment of Bacterial Lysis Efficiency and RNA Quantification

Bacterial lysis efficiency was assessed using a LIVE/DEAD™ BacLight™ assay (Thermo Fisher Scientific, cat. #L13152) to quantify bacterial viability post-lysis. A single colony of *E. coli* DH5a or *B. cereus* 569 was grown overnight in 3 mL of LB broth at 37 °C, concentrated by centrifugation at 10,000× *g* for 15 min, and resuspended in 1 mL of 0.85% NaCl according to the vendor’s guidelines. *E. coli* lysis conditions included: (1) 5 min at 60 kHz, (2) 15 min at 60 kHz, (3) 5 min at 60 kHz supplemented with 1 µg of T4 lysozyme (New England Biolabs, Ipswich, MA, USA, NEBExpress^®^ T4 Lysozyme, cat. #P8115S), and (4) 15 min at 60 kHz with 1 µg of T4 lysozyme. The piezoelectric plate was tested both inside and outside the device to evaluate its performance in different configurations. A handheld sonicator operating at 30 kHz with lysis bursts was used as a positive control, while non-lysed bacteria served as a baseline control for all experiments. Lysed and intact bacteria were stained following the vendor’s guidelines. A 2× stock solution of the LIVE/DEAD™ BacLight™ SYTO 9 and propidium iodide mixture was prepared in 5 mL of sterile water, then mixed in equal volume with the bacterial suspension in 0.85% NaCl and incubated for 15 min. The final dye concentrations were 6 µM SYTO 9 and 30 µM propidium iodide. A 5 µL aliquot of the stained bacterial suspension was placed between a slide and coverslip and imaged using an EVOS FL fluorescence microscope equipped with EVOS Light Cubes GFP and RFP. RNA concentration from lysed samples was quantified using the Qubit™ RNA High Sensitivity Assay Kit (Thermo Fisher Scientific, cat. #Q33230) and the Qubit™ 4 fluorometer.

### 2.6. Agilent Bioanalyzer Assessment of RNA Capture Pin Specificity

Following 15 min of lysis at 60 kHz with T4 lysozyme and a piezoelectric plate in contact with the bacterial suspension, an Agilent 2100 Bioanalyzer was used to assess the relative amount of 16S rRNA captured by the functionalized gold pins. The pins were immersed in the lysed bacterial solution for 2 min, then passed through a 1 mm thick PDMS sheet to remove nonspecifically attached nucleic acids. The pins were inserted into glass LightCycler^®^ Capillaries (Roche, Indianapolis, IN, USA, cat. #04929292001) containing 25 µL of RNase-free water supplemented with 80 units of RNaseOUT™ Recombinant Ribonuclease Inhibitor (Thermo Fisher Scientific, cat. #10777019). The capillaries were incubated at 60 °C for 2 min to release RNA from the pins. The extracted RNA was analyzed using the Agilent RNA Pico Kit following the manufacturer’s protocol. Data analysis was performed using Agilent 2100 Expert Software.

### 2.7. RT-qPCR Using RNA Purified from RNA Capture Pins

Functionalized gold-plated pins were used for direct RNA purification following bacterial lysis. The RCPs were inserted into the device chamber for 2 min to allow 16S rRNA hybridization to the immobilized oligonucleotides, followed by the nucleic acid release as described in the previous protocol. Experiments were conducted using *E. coli* lysates from both bacteria in suspension and those captured on the aptamer-functionalized surface of the device. Lysis was performed using a piezoelectric plate attached to the inner surface of the device for 15 min at 60 kHz with 1 µg of T4 lysozyme. The 16S rRNA was amplified using the TaqMan™ RNA-to-Ct™ 1-Step Kit (Thermo Fisher Scientific, cat. #4392653) and analyzed on a Quant Studio™ 3 PCR system. Each 20 µL reaction contained 8.5 µL of RNA sample, 10 µL of 2X RT-PCR buffer, 1.5 µL of TaqMan primers targeting a conserved bacterial 16S rRNA region (Thermo Fisher Scientific, cat. #Ba04230899), and 0.5 µL of 40X RT enzyme mix. The thermal cycling protocol consisted of reverse transcription at 48 °C for 15 min, reverse transcriptase deactivation at 95 °C for 10 min, followed by 40 cycles of PCR amplification (95 °C for 15 s, 60 °C for 1 min). A standard curve was generated using a serial dilution of *E. coli* total RNA (Thermo Fisher Scientific, cat. #AM7940) to determine the RNA concentration per RCP. Negative controls, prepared with nuclease-free water instead of RNA, were included in every experiment to monitor non-specific amplification.

## 3. Results and Discussion

### 3.1. Thiol-Conjugated Oligonucleotide Immobilization on the Gold-Plated RCPs

**Figure 2:**
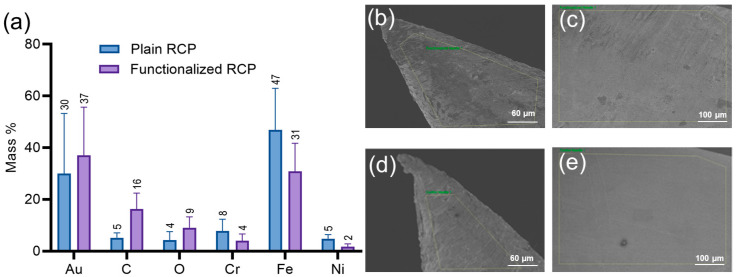
(**a**) Elemental composition of the RCP surfaces determined by SEM-EDX analysis. Each bar graph represents the average mass % of elements from five replicates, with error bars indicating the standard deviation (SD). SEM images of the tip (**b**) and body (**c**) of oligonucleotide-functionalized RCPs; SEM images of the tip (**d**) and body (**e**) of plain RCPs.

Scanning Electron Microscopy with Energy Dispersive X-ray Spectroscopy (SEM-EDX) was used to obtain surface images and determine the mass percent (% mass) of each detected element relative to the total identified mass on the RCPs. The analysis was used to estimate element distribution, confirm oligo-immobilization efficiency, and assess potential contamination. Imaging of plain and oligonucleotide-functionalized RCPs indicated that the presence of synthetic nucleic acids correlated with increased surface roughness and the appearance of artifacts and spots (Figure 2a–c). In contrast, plain RCPs exhibited a uniform and smooth surface (Figure 2d,e). Elemental analysis showed that the mass % of chromium, iron, and nickel was higher in plain RCPs. The immobilization of thiol-conjugated synthetic oligonucleotides was associated with a carbon mass % increase from 5.17% to 16.30% and an oxygen increase from 4.24% to 9.03% (Figure 2a). The detected oxygen signal was attributed to either the phosphate backbone of the oligonucleotides or surface oxidation. The average carbon-to-gold mass % ratio was used as an indirect measure to assess functionalization efficiency. This ratio was 0.17 for plain pins, while functionalized pins had a ratio of 0.44, representing a 2.6-fold increase in carbon content. The elevated carbon mass % on functionalized pins confirmed the successful attachment of oligonucleotides, while trace carbon detected on plain pins was likely due to contamination from organic compounds. The combined use of SEM imaging and EDS analysis assessed the morphological and chemical characteristics of the RCPs and confirmed the efficacy of the oligonucleotide immobilization.

### 3.2. Device Design and Aptamer-Based Selective Capture of E. coli

**Figure 3:**
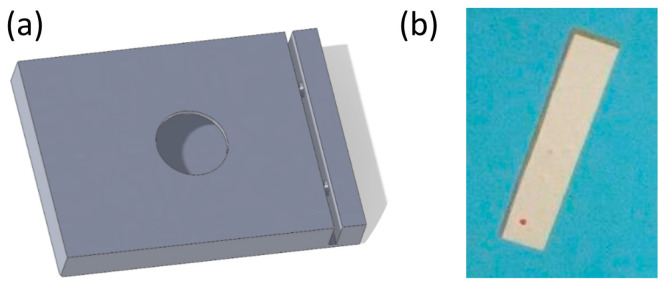
(**a**) A 3D model of the bacterial lysis platform, including the position of the piezoelectric plate on the side and the circular opening on the top for insertion of the RCPs; (**b**) The piezoelectric element used for bacterial cell disruption.

The device had dimensions of 35 mm × 28 mm × 10 mm, with a wall thickness of 0.1 mm. The bacterial lysis platform included a circular opening (15 mm in diameter) at the top for insertion of the RCPs (Figure 3a). One side of the platform was designed with an indentation that precisely matches the dimensions of the piezoelectric plate (25 mm × 5 mm × 0.30 mm providing secure placement and efficient energy transfer (Figure 3b). Upon activation, the piezoelectric plate generated acoustic waves, inducing shear forces that facilitated bacterial cell wall disruption, enabling efficient lysis.

**Figure 4:**
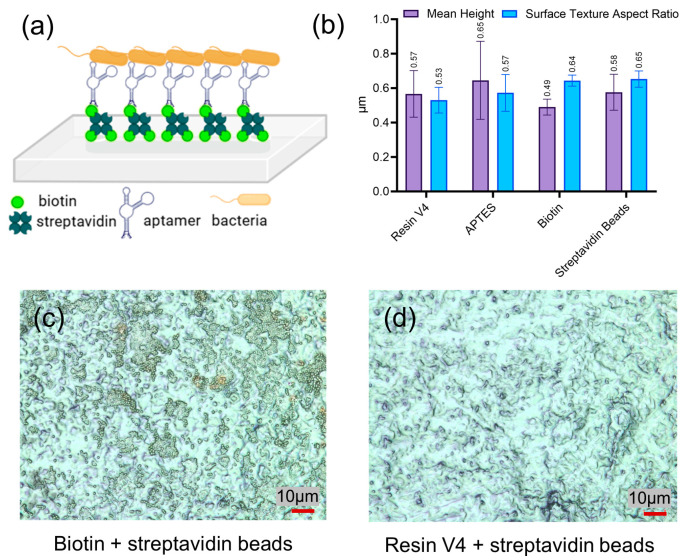
(**a**) Schematic representation of biotin-conjugated aptamer immobilization on a streptavidin-functionalized device surface. The aptamers are designed for selective binding to proteins on the outer membrane of *E. coli*. (**b**) Surface profile plot of the Resin V4 surface after sequential functionalization with APTES, biotin, and streptavidin bead obtained using laser confocal microscopy. Bar graphs represent the mean ± SD from five independent experiments. (**c**) Image of the biotin-functionalized Resin V4 surface, demonstrating the attachment of streptavidin beads. (**d**) Control experiment that assesses nonspecific binding of streptavidin to the resin surface, showing an image of the plain Resin V4 surface after streptavidin beads addition.

The lower channel wall of the platform was functionalized with biotin-conjugated aptamers to enable the selective capture of a specific bacterial strain (Figure 4a). The successful immobilization of APTES, biotin, and streptavidin was confirmed using 3D Laser Scanning Confocal Microscopy. Quantitative surface measurements were obtained from the 3D surface profile data following the addition of each layer (Figure 4b). Two key parameters were analyzed: mean height, representing the average vertical deviation and overall surface roughness, and the surface texture aspect ratio, which indicates surface anisotropy. A surface texture ratio close to 1 suggests an isotropic surface, while values approaching 0 indicate a highly anisotropic texture. The addition of APTES increased the surface roughness relative to the untreated Resin V4, likely due to the formation of an irregular polymeric layer. APTES reacts with the hydroxyl groups on the surface of the Resin V4 through a condensation reaction, forming covalent bonds between the ethoxy and the hydroxyl groups [1]. The silanization of the Resin V4’s surface results in the attachment of an amine-functionalized propyl chain to the surface, generating an amine-modified interface for the covalent attachment of biotin. Biotin immobilization resulted in a smoother surface, suggesting the formation of a uniform molecular layer that fills microscopic irregularities. This effect can be attributed to the cross-linking reaction between N-hydroxysuccinimide (NHS)-activated biotin and the amine groups of APTES, which may contribute to a more homogeneous molecular coating. Following the immobilization of streptavidin-coated beads (2.8 µm in diameter), the mean surface height increased, consistent with the successful attachment of the beads. The texture analysis indicated that the untreated Resin V4 surface and the APTES-modified surface exhibited a more isotropic texture. The addition of biotin and streptavidin beads increased surface anisotropy, likely due to the heterogeneous distribution of the biotin proteins and the larger bead structures. Surface imaging confirmed these observations. In the presence of the biotin layer, the streptavidin was covalently bound (Figure 4c). Control experiments indicated that streptavidin did not adhere non-specifically to the untreated resin surface (Figure 4d).

**Figure 5:**
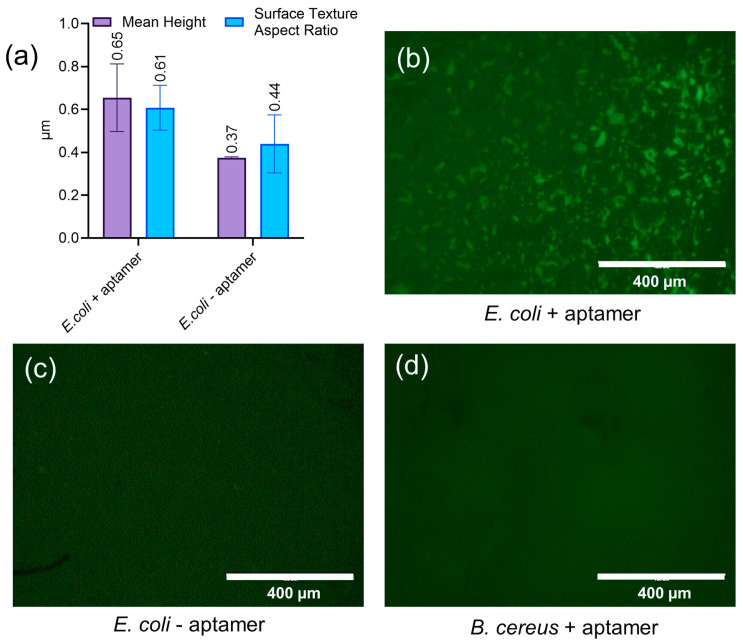
(**a**) Surface profile plot comparing *E. coli* attachment on an aptamer-functionalized surface versus a streptavidin-coated device without aptamers. (**b**) Fluorescent image of *E. coli* immobilized on the aptamer-functionalized device, visualized using FITC-conjugated aptamers. (**c**) Control experiment assessing nonspecific bacterial attachment to the streptavidin-coated surface. (**d**) Control experiment by introducing *B. cereus* to a device functionalized with *E. coli*-specific aptamers. The absence of fluorescence confirms the high specificity of the aptamers.

The selective capture of *E. coli* was evaluated using laser confocal and fluorescent microscopy. Captured bacteria were labeled with FITC-conjugated aptamers, followed by fluorescence imaging to confirm binding. To determine whether *E. coli* attachment to the lower channel wall of the device resulted from specific aptamer binding or nonspecific adherence to the device surface, the capture of bacteria was assessed under two conditions: (1) in the presence of aptamers and (2) in the absence of aptamers, where bacteria were exposed to a streptavidin-coated surface. Experiments were conducted using *B. cereus* to assess the selectivity of the aptamers. Surface roughness measurements indicated that the attachment of *E. coli* increased surface roughness and resulted in a more isotropic surface (Figure 5a). Fluorescence microscopy confirmed the selective binding of *E. coli* to the aptamer-functionalized surface, as indicated by the presence of green-labeled bacteria (Figure 5b). A fluorescence signal was not detected when *E. coli* was exposed to the streptavidin-coated surface without aptamers, demonstrating that nonspecific binding was negligible (Figure 5c). No fluorescent signal was detected when *B. cereus* was introduced to the aptamer-functionalized surface, indicating the high specificity of the aptamers for *E. coli* capture (Figure 5d). These results validate the effectiveness of the aptamer functionalization process and confirm its specificity for *E. coli* over *B. cereus* and its potential for selective bacterial capture in biosensing applications. The aptamer specifically binds to a surface-exposed *E. coli* outer membrane protein through a combination of hydrogen bonding, electrostatic interactions, and Van der Waals forces. Its secondary structural elements, including stem-loops and G-quadruplex formations, enhance both specificity and affinity, ensuring robust bacterial immobilization. This strong interaction is crucial for maximizing lysis efficiency and improving RNA yield [22].

### 3.3. Characterization of the Bacterial Lysis Efficiency of the Platform with an Integrated Piezoelectric Plate

**Figure 6:**
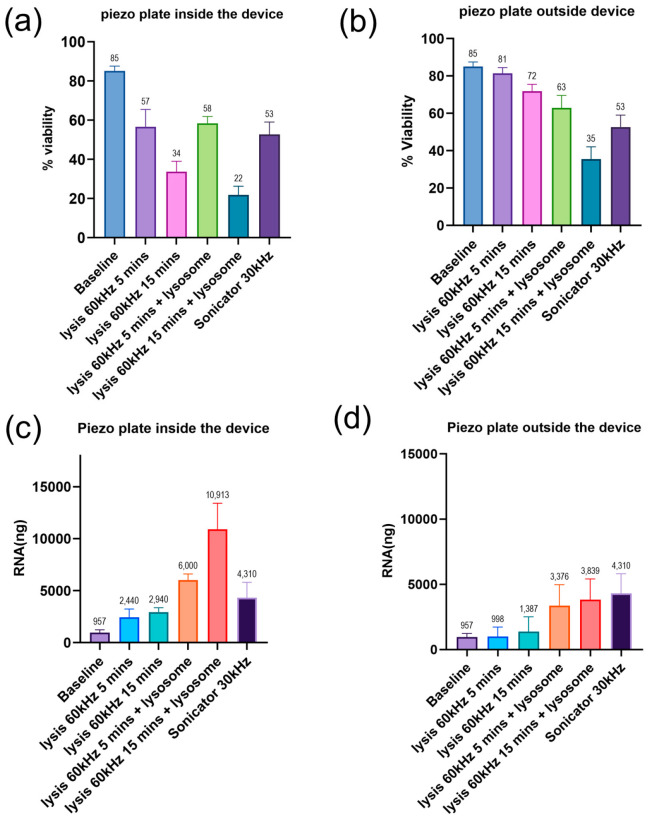
(**a**) Viability assessment of bacteria when the piezoelectric plate was in direct contact with the bacterial suspension. (**b**) Viability assessment when the piezoelectric plate was attached to the outer surface of the device. (**c**) RNA concentration was measured with the piezoelectric plate positioned inside the device. (**d**) RNA concentration was measured with the piezoelectric plate positioned outside the device. All experiments represent the mean ± SD of five biological replicates.

The bacterial lysis efficiency of the platform was evaluated at 60 kHz which was the optimal piezoelectric resonance frequency for the plate. The piezoelectric element was positioned either inside or on the outer surface of the device, and the lysis duration was varied between 5 and 15 min in the presence or absence of T4 lysozyme. The results demonstrated effective *E. coli* lysis under various conditions, with the highest efficiency observed when the piezoelectric plate was positioned inside the device and combined with T4 lysozyme. The baseline *E. coli* viability before lysis was 85.11%. When the piezoelectric plate was placed inside the device, viability decreased to 56.53% after 5 min of lysis and dropped to 33.68% after 15 min. The addition of T4 lysozyme improved the efficiency of lysis, reducing viability to 58.40% at 5 min and to 21.77% at 15 min. In comparison, conventional sonication resulted in a viability of 52.71% (Figure 6a). The attachment of the piezoelectric plate to the outer surface of the device resulted in reduced lysis efficiency. The viability was 81.39% after 5 min of lysis and decreased to 71.87% after 15 min. The presence of T4 lysozyme improved the lysis, but it remained lower than that observed when the piezoelectric plate was positioned inside the device. The bacterial viability decreased to 62.92% after 5 min and 35.45% after 15 min of lysis with T4 lysozyme (Figure 6b). These results indicate that positioning the piezoelectric plate inside the device results in higher bacterial lysis, likely due to the localized ultrasonic waves within the confined geometry of the system.

RNA concentrations were measured before and after lysis to assess the release of nucleic acid in the suspension. The average baseline RNA concentration was 957 ng. When the piezoelectric plate was positioned inside the device, the concentration increased to 2440 ng after 5 min and 2940 ng after 15 min. The addition of T4 lysozyme improved the RNA yield which was, on average, 6000 ng at 5 min and 10,913 ng at 15 min. In comparison, the sonicator treatment resulted in an RNA concentration of 4310 ng (Figure 6c). The attachment of the piezoelectric plate to the outer surface of the device resulted in lower RNA concentrations of 998 ng at 5 min and 1387 ng at 15 min. The presence of T4 lysozyme improved RNA yield; however, it remained lower than that observed when the piezoelectric plate was positioned inside the device, reaching 3376 ng at 5 min and 3839 ng at 15 min (Figure 6d). These results indicate that the combination of mechanical stress from the piezoelectric vibrations and enzymatic degradation by T4 lysozyme significantly increases bacterial lysis and the corresponding RNA release.

The developed method provides 80% bacterial lysis efficiency while requiring minimal instrumentation and eliminating the need for an amplifier. The integration of an amplifier increases the complexity of the instrumentation. However, more power and higher frequencies improve the lysis efficiency and decrease the time. Bacteria cell walls were successfully disrupted using traveling surface acoustic waves at frequencies 13 MHz and 160 MHz that resulted in 90% lysis efficiency and DNA extraction efficiency between 10 and 20% [23]. Ultrasonic lysis using piezoelectric transducers at a frequency of 130 kHz resulted in complete lysis after a few seconds with a temperature increase of only 3.3 °C. A time delay between the ultrasound burst allowed the temperature of the samples to decrease by at least 1 °C and reduce the nucleic acids and protein degradation. An increase in temperature increases the degradation of RNA [24]. Thermal lysis followed by phase-separation-based extraction of RNA and DNA in a microfluidic device resulted in a higher recovery rate than commercial column-based nucleic acid purification kits [25]. However, the thermal lysis of DNA, RNA, and proteins requires an aqueous mixture containing Triton X-100, which presents challenges for the selective capture of 16S rRNA using RCP.

The piezoelectric lysis presents several advantages over traditional mechanical lysis methods in terms of energy efficiency and scalability, making it well-suited for point-of-care applications. Piezoelectric lysis generates controlled, high-frequency vibrations that efficiently disrupt cell membranes with minimal heat production. This reduction in heat helps prevent RNA degradation, making it ideal for sensitive downstream applications. Ultrasonic lysis of *Bacillus subtilis* spores using transducers operating at a frequency of 1.4 MHz demonstrated significantly higher lysis efficiency than thermal methods [26]. Mechanical lysis, which involves physically disrupting the bacterial cell membrane using sheer force, can effectively release intracellular components. This technique often incorporates nanoscale structures within microchannels, but the applied pressure to rupture the cell wall can lead to sample fragmentation. The design of the device is critical, as the shear stress induced by these structures must be sufficient to destroy the cell membrane [27]. The incorporation of sharp nanostructures within the channels of a microdevice could increase protein yield by threefold compared to filters lacking such structures [28]. Piezoelectric techniques are more versatile and scalable, whereas mechanical lysis is limited by the design of the microfluidic device, which may not be easily adaptable for different sample types or volumes.

### 3.4. RNA Capture Pin Efficiency and Specificity

**Figure 7:**
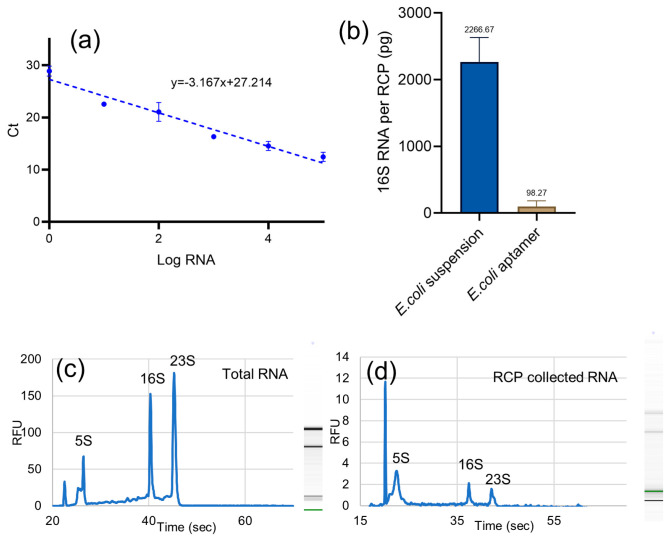
(**a**) RT-qPCR standard curve for the 16S rRNA bacterial gene using a serial dilution of total *E. coli* RNA (0–10 ng). (**b**) Average 16S rRNA concentration per RCP after RNA extraction from *E. coli* lysed in suspension or aptamer-captured to the lower surface of the device. All experiments represent the mean ± SD of five biological replicates. (**c**) Agilent 2100 Bioanalyzer electropherogram of total *E. coli* RNA. (**d**) Agilent 2100 Bioanalyzer electropherogram of RNA collected by the RCP from *E. coli* lysed in suspension, demonstrating enrichment for 16S rRNA.

The efficiency of RNA capture was assessed using direct RT-qPCR targeting the bacterial 16S rRNA gene from *E. coli* RNA extracted by the RCPs. Bacterial lysates were obtained from two conditions: suspension lysis and selective binding of *E. coli* to aptamers immobilized on the lower surface of the platform. A standard PCR curve generated using a serial dilution of total *E. coli* RNA was used to calculate the RNA concentration per RCP (Figure 7a). The results showed significantly higher RNA recovery from suspension-lysed samples compared to aptamer-functionalized surfaces. On average, RCPs extracted 2.3 ng of 16S rRNA per pin from suspension, while the aptamer-captured bacteria yielded 0.1 ng per pin (Figure 7b). This difference indicates the dependence of RCP capture efficiency on the initial RNA concentration in the sample, with bacterial suspensions providing a higher starting concentration compared to surface-captured bacteria.

The specificity of RCPs functionalized with gold-plated pins was evaluated using the Agilent 2100 Bioanalyzer. Two experiments were conducted: (1) analysis of total *E. coli* RNA and (2) assessment of 16S rRNA captured by RCPs after lysis in the device. The specificity and enrichment of 16S rRNA were determined based on the percentage of total RNA area corresponding to 16S rRNA, as analyzed by the Agilent 2100 Bioanalyzer software based on the corresponding electropherograms. The average 16S/23S rRNA ratio was 0.63 in the total *E. coli* RNA samples, indicating a higher concentration of 23S rRNA, as reflected in the larger 23S peak and increased band intensity (Figure 7c). RNA extracted using the RCPs had an average 16S/23S ratio of 1.83, representing a threefold increase in the relative abundance of 16S rRNA (Figure 7d). This enrichment of 16S rRNA over 23S rRNA supports the specificity of the capture process. One possible explanation for the presence of other rRNAs is partial sequence complementarity between the capture oligos and regions of 23S or 5S rRNA. While the oligos were designed to be specific to a conserved region of 16S rRNA, rRNA molecules share structural and sequence similarities, particularly in bacterial ribosomal complexes. This could lead to non-specific hybridization. RNA secondary structures might influence hybridization efficiency, leading to incomplete discrimination between target and non-target rRNAs [29]. Specificity can be further improved by the inclusion of competitive blocking oligos, or the use of shorter, highly selective oligonucleotide probes.

While these experiments successfully capture *E. coli* 16S rRNA and RT-qPCR amplification, RNA degradation was observed during short-term storage. Adding an RNase inhibitor immediately after purification effectively mitigated degradation; however, maintaining RNA integrity over extended storage periods remains a challenge. A study demonstrated that encapsulation significantly enhances RNA stability by protecting it from atmospheric moisture and oxygen. Stainless steel mini capsules create an anhydrous, anoxic environment that prevents RNA degradation. RNA stored under these conditions degrades much more slowly than RNA exposed to air [30]. Long-term storage of RNA was successfully performed using freeze-dry in 10% trehalose followed by storage at 4 °C [31]. Future advancements in stabilization strategies will be crucial for enhancing the storage capabilities and overall efficiency of the device for RNA-based pathogen detection and genetic analysis.

The RNA capture capacity of 0.1–2.3 ng of 16S rRNA per RCP is within the range of the detection limits for RT-qPCR. Clinical diagnostic sensitivity requirements vary depending on the infection type, bacterial load, and sample source. For some infections, bacterial loads can be as low as 1–100 CFU mL^−^^1^, requiring highly sensitive detection methods, often in the femtogram to low picogram range of bacterial RNA [32]. In contrast, for urinary tract infections, bacterial concentrations are typically higher (10^3^–10^5^ CFU mL^−^^1^), making our platform’s current sensitivity sufficient for direct detection [33].

The RNA capture pin technology represents a promising alternative to traditional RNA purification methods, offering several advantages, in terms of simplicity, speed, and minimal sample volume requirements. Unlike conventional column-based and magnetic beads-based purification methods, which often involve multiple liquid-handling steps, centrifugation, and longer processing times, the RNA capture pin technology streamlines the RNA purification process. Table 1 below summarizes the key advantages and limitations of RNA capture pin technology compared to conventional RNA purification methods, providing a detailed comparison of performance features across different platforms.

RNA capture pin technology is a simplified alternative to traditional RNA purification methods. While further optimization is needed to fully match the efficiency of established techniques, its ease of use, scalability, and minimal sample requirements support its potential for a wide range of applications.

## 4. Conclusions

RCPs for selective bacterial capture, lysis, and RNA purification. The platform demonstrated high specificity for *E. coli* capture and enabled rapid RNA isolation for subsequent amplification of the 16S rRNA gene. The results indicated that the capture efficiency of the RCPs depends on the initial RNA concentration in the sample, with higher yields observed from bacterial suspensions compared to aptamer-captured bacteria. The oligo-functionalized RCPs enrich the 16S rRNA that was successfully used for downstream genetic amplification. The integration of a piezoelectric plate within the 3D-printed device enables efficient bacterial lysis. The platform can be functionalized with aptamers to selectively bind specific types of bacteria that could provide a simplified tool for microbial diagnostics and sample preparation. The specificity of *E. coli* capture over *B. cereus* confirms the aptamer’s selectivity, demonstrating its potential for pathogen-specific applications. Future studies will focus on further validating the specificity of aptamer-based capture and amplification using mixed bacterial cultures and environmental samples. The RCP technology can be used for rapid pathogen detection, with potential applications in molecular diagnostics and microbiological research.

## Figures and Tables

**Table 1:** Summary of the key advantages and limitations of RCP technology compared to conventional RNA purification methods [34].

Feature	RNA Capture Pin	Column-Based Purification	Magnetic Beads-Based Purification
Liquid Handling	Minimal or none	Requires multiple liquid-handling steps	Requires washing and elution steps
Processing Time	Fast (a few minutes)	Moderate (30–60 min)	Moderate (30–60 min)
Sample Volume Requirement	Very low	Moderate to high	Low to moderate
Contamination Risk	Low (direct capture reduces contaminants)	Moderate (genomic DNA, protein carryover)	Low (RNA-binding surfaces)
Selectivity	High (oligo modification allows selective RNA binding)	Moderate (require DNase treatment)	High (customizable bead coatings)
Ease of Use	Very simple, with no centrifugation or complex steps	Multiple wash/elution steps	Magnet setup and multiple wash steps
Scalability	High (adaptable for field applications)	Moderate (lab-dependent)	High (automation-friendly)
Limitations	Newer technology requires further optimization	Labor-intensive	Costly, requires optimization for different applications

## Data Availability

Data are contained within this article.

## Abbreviations

The following abbreviations are used in this manuscript:

RCP	RNA capture pin
RT-qPCR	Reverse transcription-quantitative PCR
rRNA	Ribosomal RNA
